# Injuries in Artistic Gymnastics: Etiology, Prevention Strategies, and Multifactorial Perspectives—A Systematic Review

**DOI:** 10.3390/ijms262210929

**Published:** 2025-11-11

**Authors:** Raid Mekić, Vladan Milić, Oliver Radenković, Ilma Čaprić, Saša Veličković, Rifat Mujanović, Emir Biševac, Elvis Mahmutović, Zerina Salihagić, Aldina Ajdinović, Izet Kahrović, Benin Murić, Jovan Cvejić, Zoran Mojsilović, Igor Stanojević

**Affiliations:** 1 Department of Biomedical Sciences, State University of Novi Pazar, 36300 Novi Pazar, Serbia; vmilic@np.ac.rs (V.M.); icapric@np.ac.rs (I.Č.); rmujanovic@np.ac.rs (R.M.); ebisevac@np.ac.rs (E.B.); ehmahmutovic@np.ac.rs (E.M.); zsalihagic@np.ac.rs (Z.S.); aajdinovic@np.ac.rs (A.A.); ikahrovic@np.ac.rs (I.K.); bmuric@np.ac.rs (B.M.); 2 Faculty of Sport and Physical Education, University of Niš, 18000 Niš, Serbia; v.sale70@gmail.com; 3 Faculty of Sport and Physical Education, University of Novi Sad, 21000 Novi Sad, Serbia; jovancvejic96@gmail.com; 4 Faculty of Sport and Physical Education, University of Pristina in Kosovska Mitrovica, 38218 Leposavić, Serbia; zoran.mojsilovic@pr.ac.rs; 5 Department of Educational Studies Aleksinac, Academy of Educational and Medical Vocational Studies Kruševac, University of Niš, 18220 Aleksinac, Serbia; stanojevic3@gmail.com

**Keywords:** gymnastics injuries, overuse injuries, injury prevention, biomechanics, IL-6 and TNF-α, miRNA biomarkers

## Abstract

Artistic gymnastics is one of the most physically demanding sports, characterized by a high incidence of both acute and chronic injuries. Although previous research has primarily focused on biomechanical and training-related factors, the multifactorial etiology of injuries—including molecular and genetic aspects—remains insufficiently explored. This systematic review aimed to synthesize current evidence on the causes, mechanisms, and prevention of injuries in artistic gymnastics, with particular emphasis on biomechanical, molecular, and genetic determinants of injury risk and athletic performance. The review was conducted in accordance with the Preferred Reporting Items for Systematic Reviews and Meta-Analyses (PRISMA) 2020 guidelines and was registered in the PROSPERO database (Registration No: CRD420251167521). Electronic databases (PubMed, KoBSON, and Google Scholar) were searched for studies published between 2015 and 2025 using the keywords “gymnastics injuries,” “overuse injuries,” “injury prevention,” “biomechanics,” “IL-6,” “TNF-α,” and “miRNA biomarkers.” Nineteen studies met the inclusion criteria and were analyzed based on injury incidence, localization, mechanisms, and molecular and genetic associations. The majority of injuries were localized in the joints of both upper and lower extremities, particularly during puberty and at higher competitive levels. Repetitive loading, improper technique, and insufficient recovery were identified as the main etiological factors. Molecular biomarkers such as IL-6, TNF-α, and miRNAs (miR-155, miR-146a) were found to play key roles in inflammatory responses, while genetic polymorphisms including ACTN3 R577X, ESR1 rs2234693, and CYP19A1 rs936306 were associated with flexibility, explosive strength, and susceptibility to injury. Injury prevention in artistic gymnastics requires a personalized and multidisciplinary approach that integrates biomechanical, clinical, molecular, and genetic data. Incorporating molecular and genetic profiling into training and rehabilitation programs may enhance early detection of overuse conditions and optimize both health and performance outcomes in gymnasts.

## 1. Introduction

Artistic gymnastics is one of the most complex and physically demanding sports, engaging a full spectrum of motor abilities and physiological systems. Epidemiological evidence consistently shows that artistic gymnastics is among the sports with the highest injury rates in both youth and elite athletes. The annual injury incidence ranges between 1.5 and 9.2 injuries per 1000 h of training, depending on competitive level and age group, with higher rates observed in female and adolescent gymnasts [1,2]. Overuse injuries account for nearly 60–70% of all reported cases, most commonly affecting the wrists, ankles, and lower back [3,4].

Previous reviews have predominantly focused on descriptive epidemiology and biomechanical aspects of injury mechanisms in artistic gymnastics [2,5]. However, these reviews did not address molecular and genetic determinants of injury susceptibility, nor did they integrate multidisciplinary evidence combining biomechanics, inflammatory biomarkers, and genetic risk factors.

The present review therefore aims to provide a comprehensive and integrative perspective on the etiology and prevention of gymnastics-related injuries, expanding on recent biomechanical analyses of landing forces and deceleration characteristics [6]. It involves high-intensity, technically demanding tasks in which gymnasts frequently perform movements under the load of their entire body weight [7]. Due to the physical challenges, exceptional precision, and complexity of the exercises, this sport requires constant improvement of strength, flexibility, coordination, balance, and endurance. Children who begin training in gymnastics at an early age face biomechanically demanding movements on the floor and apparatus, where the upper limbs—particularly the wrist—serve as support and as tools for executing rotations, swings, and landings [7]. During periods of rapid physical growth and development, especially between the ages of 9 and 14, skeletal immaturity and biomechanical disproportions become more pronounced, thereby increasing the vulnerability of the musculoskeletal system. An imbalance between body mass and muscular strength, along with incomplete bone development, often leads to overloading of joint structures, which in turn elevates the risk of injury [8]. In this developmental stage, the biomechanical specifics of gymnastic movements, combined with intensive apparatus exercises and complex, highly rotational skills, place additional stress on the joints, particularly the wrists and ankles, which are among the most frequently injured regions in gymnasts [9,10,11]. Injuries in gymnastics can be classified as acute or chronic, and both are present to a considerable extent, especially during periods of high training intensity. Acute injuries include traumatic lesions such as fractures, sprains, and dislocations, while chronic injuries result from repetitive microtrauma that remains unrecognized or inadequately treated. One of the most common types of chronic injuries in gymnastics involves wrist injuries, which occur due to the high physical demands and repetition of movements such as rotations, landings, and swings. Within this group of injuries, overuse syndromes are particularly prominent, including ulnocarpal impaction, stress fractures of the scaphoid, and irritation of the distal radius and ulna. These often arise from improper loading, insufficient recovery, and technical errors [12]. In most cases, risk factors for injuries in gymnastics include sex, biological maturity, technical proficiency in skill execution, and weaknesses in physical conditioning, particularly inadequate prevention strategies and insufficient education of coaches and parents. Although injury prevention is based on proper education, adequate warm-up, conditioning training, flexibility exercises, and technical refinement, many injuries remain unrecognized in their early stages. This can lead to long-term complications, loss of functionality, and even premature cessation of sport participation [13,14]. While prevention and rehabilitation can be significantly improved through a more detailed understanding of biomechanical factors, the development of specific protocols for early recognition and treatment of injuries is still not being addressed comprehensively enough. The long-term effects of unrecognized or insufficiently treated injuries have a substantial impact on the later development and performance of gymnasts, underscoring the need for further research aimed at creating preventive models that can help young athletes continue their development without unnecessary risks [1,7].

It is hypothesized that injury occurrence in artistic gymnastics results from an interaction between biomechanical loading patterns, molecular responses to physical stress, and genetic predispositions.

According to the PICO framework:

Population (P): Artistic gymnasts of different age groups and competitive levels.

Intervention (I): Analysis of biomechanical, molecular, and genetic determinants of injury risk.

Comparison (C): Comparison between injured and non-injured gymnasts, as well as between sexes or training levels.

Outcome (O): Identification of key risk factors and development of personalized strategies for injury prevention and rehabilitation.

This systematic review was developed and reported in full accordance with the Preferred Reporting Items for Systematic Reviews and Meta-Analyses (PRISMA) 2020 guidelines, ensuring methodological transparency and reproducibility.

In addition to biomechanical and clinical factors, contemporary research highlights the significance of molecular and genetic determinants that can influence an athlete’s individual predisposition for certain performances, as well as the risk of injury. Genetic polymorphisms associated with muscle characteristics, flexibility, and the regulation of inflammatory processes represent a promising area for the personalization of training and prevention programs. This paper will also analyze key genetic markers linked to explosive strength and flexibility in gymnasts, thereby further enriching the understanding of the multifactorial nature of injury risk and athletic success.

This paper therefore focuses on the analysis of injury causes and the specific biomechanical factors contributing to their occurrence in gymnasts, as well as on the possibilities of prevention and rehabilitation, with particular attention to modern diagnostic methods, including molecular biomarkers of pain and inflammation, as well as genetic variants associated with injury predisposition and athletic success. By providing a detailed overview of current scientific knowledge and clinical insights, the aim is to enhance the understanding of the etiology, incidence, and biomechanical specifics of injuries, which may contribute to the development of precise guidelines for training processes and rehabilitation. This review also highlights the gaps in the literature concerning the effectiveness of preventive and rehabilitative methods, emphasizing the need for integrated and personalized protocols that can reduce long-term consequences and support the safe athletic development of both male and female gymnasts.

## 2. Materials and Methods

Focal Question

This systematic review aimed to synthesize current evidence on the etiology, prevention, and molecular determinants of injuries in artistic gymnastics. The main research question was formulated according to the PICO framework:

Population: Artistic gymnasts (youth and elite, male and female)

Intervention/Exposure: Gymnastics training and performance-related activities

Comparison: Training load, biomechanical, and molecular parameters

Outcome: Injury incidence, type, risk factors, and molecular/biomechanical correlates

Language: Only studies published in English were included in the review.

Databases and Search Strategy

The review was conducted in accordance with the PRISMA 2020 guidelines [15] and was registered in the PROSPERO database (Registration No: CRD420251167521).

A systematic literature search was performed using the electronic databases KoBSON, PubMed, and Google Scholar, covering the period from 2015 to 2025.

The selected time frame was chosen to focus on recent literature integrating molecular, genetic, and biomechanical perspectives, as these research domains have expanded significantly over the past decade, providing more comprehensive insights into injury mechanisms and prevention strategies in artistic gymnastics. The following keywords were used: “Gymnastics injuries,” “Overuse injuries,” “Injury prevention,” “Biomechanics,” “IL-6 and TNF-α,” and “miRNA biomarkers.”

Keywords were combined using logical operators (AND, OR), and search strategies were adapted to the specific features of each database to maximize both sensitivity and relevance.

Manual screening of reference lists from relevant review and original research articles was also conducted to identify additional eligible studies.

Study Selection: Titles and abstracts were screened to determine eligibility.

Inclusion criteria: Studies addressing injuries in the context of artistic gymnastics (including causes, prevention, molecular biomarkers, genetic variants, and biomechanical specifics);

Empirical research (prospective/retrospective studies, cross-sectional studies, clinical trials);

Peer-reviewed journal articles with full-text availability in English.

Exclusion criteria:

Studies unrelated to artistic gymnastics;

Non-injury-related papers;

Commentaries, letters, or papers with unclear methodology;

Duplicates;

Studies without full-text availability.

A preliminary search yielded 88 studies. After removing duplicates and non-eligible titles, 46 were excluded. Further evaluation excluded 4 studies not in English, 8 not meeting inclusion criteria based on abstracts, and 11 due to inappropriate study design.

In total, 19 studies were included in the final analysis.

**Figure 1:**
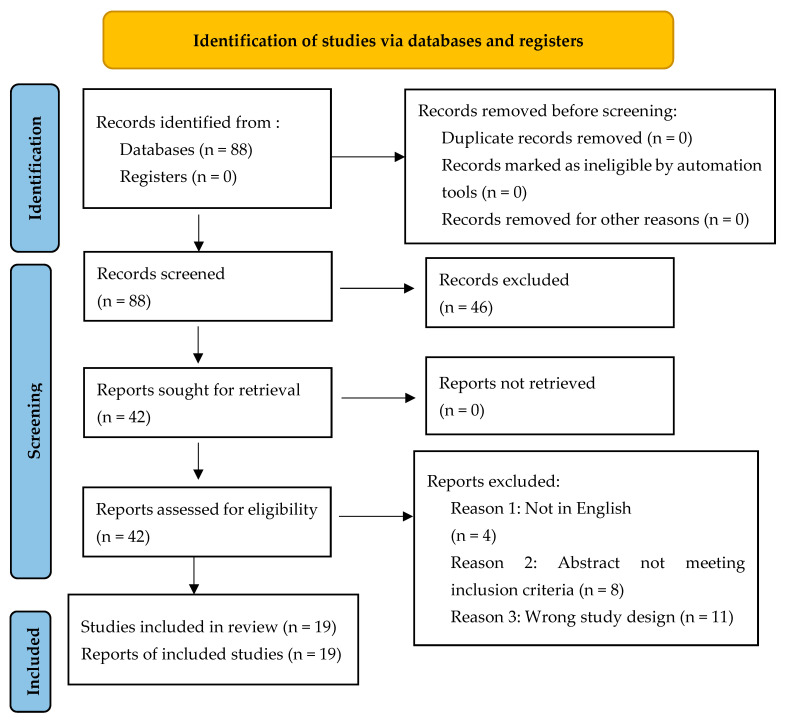
PRISMA 2020 flow diagram of the study selection process.

The study selection process is illustrated in Figure 1 (PRISMA flow diagram).

Data Extraction

The following data were extracted from each study: author(s), publication year, participant characteristics, study aim, main findings, and conclusions.

Data Analysis

A descriptive synthesis was conducted to summarize findings regarding injury prevalence, anatomical location, and underlying mechanisms in artistic gymnastics.

Quality Assessment

The risk of bias was assessed using the Newcastle–Ottawa Scale (NOS) for observational studies [16]. Two independent reviewers (R.M. (Raid Mekić) and O.R.) conducted the assessment, with discrepancies resolved by consensus or by a third reviewer (I.Č.).

The NOS scores ranged from 7 to 9, indicating good methodological quality across included studies. Details of the scale and scoring criteria are provided in Appendix A (Table A1).

## 3. Results

A total of 19 studies met the inclusion criteria and were incorporated into this systematic review. These studies were conducted across various countries and examined different aspects of injuries in artistic gymnastics. The study selection process, from identification to final inclusion, is illustrated in Figure 1.

### Study Characteristics

The included studies encompassed gymnasts of diverse ages, sexes, and competitive levels. Overall, younger athletes were more prone to upper extremity injuries, whereas older gymnasts more frequently experienced overuse-related conditions.

With respect to sex-specific patterns, female gymnasts were more often affected by injuries to the joints, knees, and elbows, while male gymnasts predominantly sustained injuries of the shoulders, hands, and forearms. Moreover, a higher competitive level was consistently associated with an increased risk of more severe and chronic injuries.

The most commonly reported injury mechanisms included contact with the apparatus or surface, repetitive movements, and excessive loading. Although the use of braces and rehabilitation strategies showed certain benefits, general prevention programs alone proved insufficient unless combined with targeted load management.

Detailed information regarding study design, participant characteristics, injury types, and main findings is presented in Table 1.

**Table 1:** Overview of studies on the incidence, localization, and risk factors of injuries in artistic gymnastics.

Authors	Sample (*n*)	Research Aim	Main Findings	Conclusion
Westermann, Giblin, Vaske, Grosso, & Wolf (2015) [17]	*n* = 119 (64 M, 55 F)	To determine differences in the mechanism and incidence of injuries between male and female gymnasts	24.4% of injuries in females required surgery compared to 9.2% in males; 55% of injuries in females affected the upper extremities, 62.6% in males; 31% of injuries in females affected the lower extremities, 27% in males.	Incidence and localization of injuries were similar in both sexes, but females sustained more severe injuries.
McLaren et al. (2015) [18]	*n* = 50	To examine the impact angle during back handspring and its association with wrist pain	Impact angle during back handspring was 95°. Wrist pain was reported by 15 participants. Shoulder angle and years of training correlated with wrist impact angle.	Participants with greater shoulder angle were at higher risk of wrist pain.
Saluan, Styron, Ackley, Prinzbach, & Billow (2015) [19]	*n* = 823, 13–26 g	To investigate the frequency of injuries over a 21-year period	833 (22.53%) upper extremity injuries, 2242 (60.91%) lower extremity injuries; shoulder 4%, back 11%, head/neck 1.2%	A higher number of injuries were recorded in gymnasts at higher competitive levels, and vice versa.
Guerra et al. (2016) [20]	*n* = 19	To investigate the prevalence of wrist pain in gymnasts	Wrist pain prevalence was 82%, bilateral in 53%. 47% of participants were limited in performance; 82% reported pain on pommel horse, 17% on floor, 12% on parallel bars.	Wrist injuries are frequent and significantly affect gymnasts’ performance.
Rodríguez-Camacho et al. (2016) [21]	*n* = 14 (F), 14–22 g	To determine injury incidence in female gymnasts from Bogotá during one season	Elbow 24%, wrist 19.5%, ankle 21%; sprains were the most common injury mechanism (31.6%)	Women’s artistic gymnastics is a sport with a high injury rate.
Goulart et al. (2016) [22]	*n* = 20 (M), 23.1 ± 6.5 g	To investigate the incidence of injuries in elite male gymnasts	Forearm 36%, ankle 16.2%, hands and fingers 14.4%, shoulder 12.6%, lower back 9%, wrist 8.1%, knee 5.4%	Most injuries in men’s gymnastics are caused by overtraining on apparatus such as floor, pommel horse, and rings.
Boucher et al. (2017) [23]	*n* = 1	To evaluate the effect of a rehabilitation program on wrist injuries	Significant improvement in strength and motor control. The program enabled return to gymnastics activities.	The rehabilitation program resulted in positive outcomes and successful return to gymnastics.
Trevithick et al. (2018) [24]	*n* = 399	To examine methods of preventing wrist pain in gymnasts	Gymnastics wrist supports were recommended as the primary method of wrist pain prevention.	Supports are effective preventive measures for reducing wrist pain.
Kox et al. (2018) [25]	-	To analyze factors contributing to overuse wrist injuries	Identified 17 signals and three limiting factors indicating overuse wrist injury.	Risk factors contributing to increased incidence of wrist injuries were identified.
Ashwell et al. (2019) [26]	-	To examine the effects of immobilization and rest on wrist injuries	Wrist immobilization, rest, and a delayed return to sport.	Return to sport is possible after appropriate rehabilitation and rest.
Trevithick et al. (2019) [27]	*n* = 48	To assess the effects of wrist supports on joint pain in gymnasts	Significant reduction in pain while using supports, particularly on pommel horse, floor, and parallel bars.	Supports significantly reduce wrist pain and improve performance.
Paxinos et al. (2019) [28]	*n* = 156 (116 F, 40 M), 14–36 g	To examine injury incidence in elite gymnasts over a 10-year period	Hip 18.5%, ankle 16.5%, lumbar spine 16%, foot 16%; most common diagnoses: tendinitis 32%, low back pain 20%, sprains 12%	Injuries are frequent in this sport, with 9% requiring surgery.
Chandran et al. (2021) [29]	*n* = 1200 (742 F, 458 M), 13–31 g	To examine injury incidence in gymnasts at the national level (2014–2019)	Knee 13.1%, ankle 12.6%, foot 12.1% in females; lower leg 11.6%, head/face injuries 10.4% in males.	Injuries are more frequent during competition (35.7%) than training, and injury incidence increases with competitive level.
Kruse, Nobe, & Billimek (2021) [30]	*n* = 2102	To examine the incidence of injuries in gymnasts during competitions (2008–2018)	50% of injuries affected the lower extremities, 24% the upper extremities, 17% the head; the most common injury mechanism was contact with the surface (66%).	Achievement motivation is considered a risk factor for injuries due to gymnasts performing elements beyond their technical capacity.
Farì et al. (2021) [31]	*n* = 79 professional gymnasts (artistic and rhythmic gymnastics)	To investigate the prevalence and risk factors for musculoskeletal pain in professional gymnasts	82.3% of athletes reported musculoskeletal pain. Significant associations were found between pain and: training duration (*p* = 0.041); right wrist pain and BMI (*p* = 0.001); hip pain and BMI (*p* = 0.030); right wrist pain and age (*p* = 0.038); left wrist pain and age (*p* = 0.004); right shoulder pain and age (*p* = 0.035); time spent sitting and pain incidence (*p* = 0.045).	Prolonged engagement in gymnastics, higher BMI, older age, and prolonged sitting increase the risk of musculoskeletal pain. Preventive strategies are needed to safeguard athletes’ health.
Tisano, Zynda, Ellis, & Wilson (2022) [32]	*n* = 34,000, >7 years	To analyze data from the National Electronic Injury Surveillance System on injury rates among male and female gymnasts	Ankle 12.19%, wrist 8.33%, knee 11.5%, fingers 3.88%, neck 3.01%, shoulder 9.08%	In adolescence, males had higher injury rates in the ankle and wrist, whereas females had higher rates of upper extremity injuries.
Sastre-Munar et al. (2022) [33]	*n* = 160 (89 F, 51 M)	To determine injury rates in gymnasts across different competitive levels	Ankle 25.5% of all injuries, knee 14.2%, lower back 10.4%	Injuries are more frequent at higher competitive levels.
Pei et al. (2023) [34]	*n* = 1115 adolescent females (518 with dance-related and 597 with gymnastics-related injuries)	To determine whether there are differences in musculoskeletal injury patterns between adolescent girls involved in dance and gymnastics	Most frequent injuries in dance were sprains/strains (33.3%), while in gymnastics fractures predominated (37.3%). In gymnastics, the likelihood of sprains was 74% lower than in dance, but the likelihood of fractures was 3.84 times higher.	Dance is associated with more frequent sprains, whereas gymnastics carries a higher risk of fractures. Differences in injury patterns may inform more sport-specific prevention planning.
Gram et al. (2025) [35]	*n* = 119 gymnasts in the intervention group (IG) and *n* = 86 in the control group (CG)	To evaluate whether a specific injury prevention program (IPP) reduces the incidence of overuse injuries in the knees, lower back, and hips/groin in competitive female gymnasts compared with usual training	- Injury incidence measured monthly using OSTRC-H2-Response rate to OSTRC-H2: 94% - No difference in injury incidence between groups: odds ratio = 0.86 (95% CI 0.32–2.29); *p* = 0.77 (intervention vs. control group)	A specific injury prevention program (IPP) focusing on exercises to improve physical capacities, strength, flexibility, and movement control, was not sufficiently effective in reducing the incidence of injuries at targeted sites.

Legend: *n*—number of participants; M—male; F—female; OSTRC-H2 (Oslo Sports Trauma Research Center Questionnaire on Health Problems).

## 4. Discussion

In line with the objectives of this study, the results of the analysis presented in Table 1 indicate not only the incidence and causes of injuries among gymnasts, but also the potential of modern diagnostic methods for improving prevention, including both biomechanical and molecular approaches [1,7]. This integrative perspective represents a novel contribution of the present review, as previous studies have rarely combined biomechanical determinants with molecular and genetic markers (IL-6, TNF-α, miRNA, ACTN3, ESR1, CYP19A1) in the context of artistic gymnastics injuries. From a clinical perspective, the findings of this study provide valuable insights for therapists and injury prevention specialists in artistic gymnastics. These results enable the development of specific rehabilitation programs that take into account the biomechanical demands of the sport, as well as personalized preventive strategies. Understanding the specific mechanisms of injury—particularly in relation to jumps, landings, and dynamic movements characteristic of gymnastic disciplines—is crucial for reducing injury incidence. However, the interpretation of existing findings, both from previous research and from the current analysis, should be approached with caution due to methodological limitations. Most of the reviewed literature does not clearly distinguish between acute and chronic injuries, which significantly complicates the differentiation of approaches in injury prevention and rehabilitation in gymnastics [5]. This missing distinction has direct implications for the design of specific preventive strategies and rehabilitation plans that could be more effective in reducing injury rates. For example, acute injuries that occur as a result of immediate impacts or improper execution of exercises, such as strains or sprains, require rapid and targeted intervention, whereas chronic injuries, which develop gradually due to repetitive movements and stress, demand long-term rehabilitation and adjusted training regimens. Longitudinal monitoring of young gymnasts, with particular emphasis on biomechanical movement analysis, represents a key avenue for future research. Evaluating the effects of different preventive strategies and the role of individual factors—such as body composition, flexibility, previous injuries, and biomechanical specifics (e.g., landing forces)—may contribute to a better understanding of how these factors influence injury risk. Such research would enable the development of more precise preventive and rehabilitative strategies by linking biomechanical load profiles with specific molecular responses (e.g., inflammatory cytokine expression and muscle repair markers). Tailoring these strategies to the distinct technical and physical demands of each gymnastics discipline could significantly enhance injury prevention and recovery efficiency.

### 4.1. Causes

Injuries in artistic gymnastics are a frequent occurrence, primarily due to the exceptionally high biomechanical demands of the discipline, which require precise coordination, strength, flexibility, and the execution of complex technical elements. Numerous clinical studies indicate that stress fractures of the scaphoid bone, epiphyseal injuries, and ligamentous lesions are among the most common diagnoses, particularly in younger gymnasts during periods of intensive growth [36,37]. The causes of these injuries are largely multifactorial, with excessive and repetitive mechanical loading, early sports specialization, and insufficient biomechanical alignment between the musculo-tendinous and skeletal systems standing out as key contributors [26,38].

During the pubertal growth spurt, particularly between the ages of 10 and 14, imbalances in the development of the muscular and skeletal systems render the epiphyses of the distal radius especially vulnerable [39,40]. This vulnerability is further exacerbated by elements involving high axial loading, such as exercises on the floor and pommel horse, which according to [20], cause wrist pain in as many as 82% of gymnasts.

The literature also points to a higher incidence of severe injuries among female gymnasts compared to their male counterparts, which may be attributed to biomechanical and physiological sex differences. Ref. [17] reported that females had a higher rate of severe injuries (24.4%) compared to males (9.2%), with females more frequently injuring the upper extremities, whereas males predominantly sustained lower extremity injuries. Age and competitive level further influence injury incidence, with gymnasts over the age of 14 more likely to exhibit symptoms of chronic overuse [28,30].

### 4.2. Prevention

In the prevention of injuries in gymnastics, it is important to take into account the specific challenges faced by gymnasts, as well as relevant preventive strategies applied in other sports such as football and basketball. Although gymnasts are prone to lower extremity injuries similar to athletes in team sports, they also face unique risks, such as injuries to the wrists, shoulders, and back. The complex movements characteristic of gymnastics, such as acrobatics, apparatus balancing, and jumping, require particular attention in injury prevention, given the strain they place on these specific body regions [41].

The results and contemporary recommendations on injury prevention in sports clearly indicate the need for systematic programs that include specific exercises for stabilization, strengthening, and technical correction, which is particularly important in gymnastics due to its complex biomechanical demands. In this context, the case report of a young gymnast with a wrist injury is especially noteworthy, as he was successfully rehabilitated through a progressive program focused on identified musculoskeletal deficits and technical errors in performance [23]. The program lasted eight weeks and consisted of a total of 11 therapy sessions, divided into three progressive phases. In the acute phase, the focus was on reducing pain and inflammation using cryotherapy and manual therapy, along with the introduction of non-weight-bearing mobility exercises. Targeted muscle groups of the hand and forearm were activated, while breathing and core stabilization exercises were also incorporated. In the recovery and movement control phase, progressive strengthening exercises for the shoulder girdle and proprioceptive balance training were introduced, together with the simulation of gymnastic movements under reduced load. Emphasis was placed on learning proper support technique and correcting joint positioning. In the functional reintegration phase, specific gymnastic elements were gradually reintroduced under controlled conditions, with a focus on coordination, explosiveness, and technical correction. Throughout the entire process, functional assessment tools were employed, including subjective pain scales, muscle strength and motor control tests, as well as video motion analysis for objective evaluation of progress [23]. This case clearly demonstrates the importance of integrating principles such as correct biomechanics of movement [42], strengthening of core and upper extremity stabilizers [43], and the education of athletes and coaches in injury prevention and the early recognition of overuse symptoms [25,44]. The application of such a rehabilitation protocol in practice directly contributes to achieving the goals of injury prevention highlighted in the literature [31,41], emphasizing the need to include not only strength and flexibility exercises in daily training, but also education on technical details, proper load management, and risk control. In this way, the experience from this case report not only confirms theoretical guidelines but also provides a model for practical application that may serve as a foundation for future preventive and rehabilitative protocols in gymnastics. Research on preventive programs in sports such as football highlights the importance of muscle strengthening, improved coordination, core stabilization, and increased flexibility. Programs such as FIFA 11+, which have proven effective in preventing lower extremity injuries, can be adapted to gymnastics, but with an emphasis on the body regions most at risk for gymnasts, such as the shoulders and wrists [43].

Additionally, analyses of postoperative recovery in elite athletes provide important insights that can be transposed to the context of gymnastics. For example, a study on anterior cruciate ligament (ACL) reconstruction in professional soccer players showed that as many as 92.6% of athletes returned to play [45], with an average recovery time of 256 days. Rehabilitation was carried out through a multidisciplinary approach focusing on proprioception, muscle strengthening, and biomechanical correction of movement [46]. Similarly, in cases of Achilles tendon rupture in the NBA, NFL, and MLB leagues, approximately 69% of athletes successfully returned to competition, with rehabilitation protocols including controlled loading, eccentric exercises, and functional training [47,48].

For severe hamstring injuries, such as proximal hamstring avulsions, studies report high rates of return to previous levels of athletic performance, particularly when individualized progressive programs emphasizing pelvic stabilization and muscle balance are applied [49].

Molecular mechanisms play a key role in understanding the regeneration of muscle and connective tissue, particularly through the action of satellite cells and the regulation of inflammatory cytokines, which significantly influence the effectiveness of recovery after injury [31,50]. These findings highlight the potential application of molecular biomarkers in individualized monitoring of rehabilitation progress in gymnasts.

These data clearly indicate that precisely planned and targeted rehabilitation protocols, incorporating core stabilization, neuromuscular control, and functional movement patterns, can form the basis for developing prevention and rehabilitation strategies in gymnastics. Proper biomechanics of movement also play a crucial role in injury prevention. For example, the study by [42] demonstrated the importance of proper landing technique in sports such as basketball. This principle can also be applied to gymnastics, where emphasis should be placed on controlled execution of acrobatics and landings, thereby reducing stress on key joints such as the knees, elbows, and shoulders. All of this underscores the importance of integrating principles of strength, stabilization, and biomechanical precision into gymnasts’ training processes, both in prevention and rehabilitation [51].

In addition to physical exercises, the education of coaches and athletes is of key importance. Coaches should be trained to properly implement preventive exercises and correct technical errors, while athletes should be continually educated on the importance of correct technique, training load, and injury prevention [52]. Specific rehabilitation protocols addressing ligament, tendon, and overuse injuries are also necessary to minimize the negative consequences of injuries [32]. Studies such as [25] identify specific symptoms of overuse that may indicate early signs of wrist injuries. Regular equipment checks and proper apparatus setup further reduce injury risk [44]. The use of orthoses and braces to decrease axial pressure on the joints during high-intensity dynamic elements has proven highly effective in injury prevention [24,25]. Injuries are more frequent during competitions, when physical demands and psychological pressure are at their peak. According to [29], as many as 35.7% of injuries occur during competition, highlighting the need for careful planning of recovery, periodization, and load management. Developing preventive programs that address both physical and psychological aspects—such as recovery strategies and mental preparation—can substantially contribute to reducing injury rates [35].

In conclusion, injury prevention in gymnastics requires a comprehensive approach that encompasses physical health, exercise technique, training load and intensity adjustments tailored to individual athletes’ needs, core stabilization, as well as athlete and coach education [34]. Integrating preventive programs into gymnasts’ daily training—programs that emphasize strength development, stabilization, and technique correction—can significantly reduce injury risk and enable athletes to achieve top-level results without compromising their health [53].

### 4.3. Molecular Biomarkers of Pain and Inflammation: New Directions in Personalized Injury Prevention in Gymnastics

Although most previous research on injuries in artistic gymnastics has predominantly focused on biomechanical, functional, and training-related aspects, increasing attention is being given to the molecular dimension of tissue responses to physical stress, with details on key biomarkers of pain and inflammation presented in Table 2. Microtraumas resulting from repetitive mechanical loading trigger a cascade of cellular signals involving inflammatory cytokines, regulatory miRNAs, and signaling pathways such as NF-κB and JAK/STAT, making them critical targets in contemporary research on prevention and recovery.

Interleukin-6 (IL-6) and tumor necrosis factor alpha (TNF-α) represent two central proteins in both systemic and local inflammation induced by physical exertion. These cytokines not only modulate the immune response but also directly affect the regeneration of muscle and connective tissue, as well as the subjective perception of pain. Elevated expression of IL-6 and TNF-α is often recorded following intensive training, particularly in athletes engaged in highly explosive disciplines such as gymnastics, and is associated with prolonged recovery and reduced functionality [54,55].

In addition to proteins, micro-RNA molecules (miRNAs)—small non-coding RNA transcripts that regulate gene expression post-transcriptionally—also play a significant role in inflammation and tissue repair. For example, miR-155 exerts a pronounced pro-inflammatory effect by stimulating the production of IL-6 and TNF-α through activation of the NF-κB pathway, which may contribute to the development of chronic inflammation in athletes exposed to cumulative loading [56]. In contrast, miR-146a has an anti-inflammatory effect, inhibiting the proteins IRAK1 and TRAF6, thereby reducing excessive inflammatory responses and supporting tissue homeostasis [57,58]. This balance between “pro-inflammatory” and “protective” miRNAs may be crucial in modulating the response to microtraumas.

Current research also indicates specific changes in miRNA expression that depend on the intensity, duration, and type of physical load. Molecules such as miR-23b-3p and miR-181 are associated with the regulation of regenerative and inflammatory processes in muscles and tendons, while miR-206 and miR-499 represent potential markers of muscle damage and adaptation [59,60]. Changes in circulating miRNAs have been observed even after single bouts of exercise, highlighting their potential for real-time monitoring of athletes’ physiological status.

Another important group of molecules are heat shock proteins (HSPs), particularly HSP70 and HSP90, which act as cellular “protein guardians.” Their increased expression following exertion protects cells from stress, reduces oxidative damage, and accelerates recovery, making them additional candidates for biomarkers of adaptation and overuse [50].

The integration of these biomolecular indicators into sports medicine protocols could significantly enhance the personalization of training processes, enable early detection of overuse, and support the optimization of rehabilitation. It is recommended that future research include the analysis of serum concentrations of IL-6 and TNF-α using ELISA methods, as well as the quantification of key miRNA expression (e.g., miR-155, miR-146a, miR-23b-3p, miR-181) through qRT-PCR techniques. In this way, it would be possible to develop early-warning biomarkers that could play a crucial role in preventing more severe injuries, as well as in assessing athletes’ readiness to return to training and competition [50,61]. From a practical perspective, these biomarkers could become an integral part of athlete monitoring and load management systems in gymnastics. Regular assessment of cytokine concentrations and miRNA expression profiles would allow for the early detection of physiological stress and tissue overload, even before the onset of clinical symptoms. Such information could support coaches and medical teams in adjusting training intensity, scheduling recovery periods, and applying individualized preventive or rehabilitative interventions. In this way, molecular monitoring would bridge the gap between laboratory research and everyday practice, enabling a more precise and personalized approach to injury prevention and recovery.

Moreover, the personalization of preventive and rehabilitative strategies in artistic gymnastics should be guided by a multifactorial profile that includes biomechanical, physiological, and genetic determinants. Biomechanical assessment enables the identification of movement inefficiencies and joint overload patterns that predispose athletes to specific injury types. Training history—including load volume, intensity, recovery cycles, and exposure to specific apparatus—provides essential information for understanding cumulative stress and adaptive capacity. Finally, genetic predispositions, such as variations in genes related to collagen synthesis (e.g., *COL1A1*, *COL5A1*) or inflammatory regulation (*IL-6*, *TNF* polymorphisms), may modulate individual susceptibility to injury and recovery potential. Considering these factors together would allow for the development of more accurate, athlete-specific models of injury prediction, prevention, and rehabilitation in gymnastics.

**Table 2:** Overview of key biomarkers of pain and inflammation relevant to sports injuries and microtraumas in gymnastics.

Biomarker	Molecule Type	Signaling Pathway/Target Function	Role in Load and Microtrauma	Detection Method	Reference
IL-6	Citokin	Activates JAK/STAT, regulates metabolism and muscle adaptation	↑ after training; indicator of inflammation, regeneration, and fatigue	ELISA	Pedersen & Febbraio (2008) [54] Suzuki et al. (1999) [55]
TNF-α	Citokin	Activates NF-κB, MAPK pathways	Induces pain, protein breakdown, prolongs inflammation and delays regeneration	ELISA	Suzuki et al. (1999) [55] Leuchtmann et al. (2021) [50]
miR-155	miRNA	Enhances IL-6, TNF-α expression via NF-κB activation	Pro-inflammatory effect, ↑ during overuse; potential indicator of chronic inflammation	qRT-PCR	O’Connell et al. (2010) [56]
miR-146a	miRNA	Inhibits IRAK1 and TRAF6, negative regulation of NF-κB pathway	Anti-inflammatory; protects against excessive inflammation, potential biomarker of resilience	qRT-PCR	Taganov et al. (2006) [57] Lee et al. (2016) [58]
miR-125	miRNA	Regulates TLR signaling expression	Maintains balance between inflammation and immunosuppression	qRT-PCR	Lee et al. (2016) [58]
miR-23b-3p	miRNA	Regulates regeneration and muscle proliferation	Increases after adaptation to exercise; linked to tendon and muscle tissue recovery	qRT-PCR	Ramos et al. (2018) [59] Podgórska et al. (2024) [60]
miR-181	miRNA	Regulates immune tolerance and regeneration	Expression changes associated with adaptation to intensive training	qRT-PCR	Ramos et al. (2018) [59]; Podgórska et al. (2024) [60]
miRNA-206	miRNA	Specific to muscle regeneration, involved in myogenesis	Useful for assessing muscle damage after training or injury	qRT-PCR	Podgórska et al. (2024) [60]
miRNA-499	miRNA	Cardiac and skeletal muscle-specific	Acts as a marker of muscle damage and systemic stress	qRT-PCR	Ramos et al. (2018) [59]
HSP (HSP70, HSP90)	Heat Shock Proteini	Responds to stress, stabilizes proteins, accelerates recovery	↑ during muscle damage and inflammation; potential adaptive markers	Western blot, ELISA	Leuchtmann et al. (2021) [50]

Legend: ↑ indicates an increase compared to baseline values.

### 4.4. Genetic Variants and Predisposition to Athletic Performance and Injuries

In addition to inflammatory biomolecules activated after physical stress, genetic variants represent an important component of the molecular profile of athletes, as they can predetermine abilities in specific disciplines as well as injury risk. In contemporary sports science, increasing attention is devoted to genetic factors that may contribute to a better understanding of individual differences in athletic abilities, responses to training loads, and injury predisposition. Genetic variants, along with hormonal and biochemical markers, constitute a key part of the molecular profile of athletes, enabling a personalized approach to training processes and the selection of sporting talent. Details on key genetic markers associated with performance and flexibility in gymnastics are presented in Table 3.

One of the most extensively studied genetic markers in the context of athletic performance is the ACTN3 R577X polymorphism, which influences the presence of the functional protein alpha-actinin-3, characteristic of fast-twitch (type II) muscle fibers. The presence of the R allele is associated with greater muscular strength and explosiveness, as confirmed by numerous studies. A particularly significant correlation has been observed between the R allele and higher D-scores in disciplines requiring explosive power, such as floor exercise, rings, and vault [62,63]. Furthermore, the frequency of this allele has been found to be higher in gymnasts competing at higher levels, which further supports its role in achieving elite athletic performance [64,65].

In addition to strength, flexibility represents a key component in the successful execution of apparatus elements that require a high degree of mobility, such as pommel horse, parallel bars, and horizontal bar. In this context, polymorphisms in the ESR1 (rs2234693) and CYP19A1 (rs936306) genes play a significant role. The presence of the C allele of ESR1 and the T allele of CYP19A1 has shown a positive correlation with greater flexibility and better performance on apparatus requiring a high level of mobility [66,67]. These genetic variants influence aromatase enzyme activity and estrogen receptor expression, which may contribute to greater connective tissue elasticity and improved biomechanical characteristics of the musculoskeletal system.

Interestingly, the aforementioned genetic variants have not demonstrated a significant association with athletic performance in female gymnasts, which may be explained by higher concentrations of estradiol that potentially mask the effects of individual SNP variants [68,69]. This difference underscores the need for a sex-specific approach when interpreting genetic findings in a sports context.

The contribution to understanding biological adaptation to load does not come exclusively from genetic studies but also from research on biomarkers of the response to physical stress. In this regard, the study by [70] is particularly noteworthy, as it analyzed bone remodeling markers following acute anaerobic load during the Wingate test (WAnT) in elite gymnasts (EG) and physically active men (PAM). The results showed that the concentration of the bone resorption marker CTX did not significantly change in gymnasts after the WAnT test, whereas in physically active men, a marked increase was recorded. These findings suggest that long-term specialization in gymnastics induces adaptive changes that protect bone tissue from excessive resorption triggered by acute physical stress [71].

Furthermore, upper-extremity strength (measured using the WAnT arm test) was significantly higher in gymnasts, while no differences were observed in lower-extremity strength, further emphasizing the specific morpho-functional profile of gymnasts. Interestingly, the increase in CTX was observed exclusively in non-specialized physically active individuals, suggesting that local muscle strength is not the only factor influencing bone response, hormonal and genetic factors also play an important role.

Within the same study, vitamin D status was also examined, with lower concentrations of 25(OH)D and 24,25(OH)_2_D_3_ being associated with higher resting CTX levels. This implies that inadequate vitamin D status may contribute to increased bone resorption even in physically active individuals, since the active form of the vitamin, 1,25(OH)_2_D_3_, directly influences osteoclast and osteoblast function [66,72]. Moreover, although the metabolite 24,25(OH)_2_D_3_ has traditionally been considered inactive, recent research suggests its role in regulating bone healing and balancing the potentially toxic effects of the active vitamin D form.

In this sense, the integration of genetic information with biomarkers of inflammation and hormonal status (e.g., IL-6, TNF-α, miRNA, vitamin D) can significantly contribute to the development of personalized training protocols, load optimization, and early identification of athletes at increased risk of injury. The introduction of genetic profiling into sports practice opens new possibilities for individualized prevention, talent selection, and long-term health preservation of athletes, particularly in highly demanding disciplines such as artistic gymnastics.

**Table 3:** Key genetic markers associated with performance and flexibility in gymnastics.

Marker Type	Gene/Molecule/SNP	Biological Function/Role	Association with Abilities/Adaptation	Specificity in Gymnastics	Reference
Genetic	ACTN3 (R577X)	Alpha-actinin-3, component of type II fibers	R allele is associated with explosive strength and fast muscle contractions	Higher D-scores in floor exercise, vault, and rings	Hassan & Abdalkarim, 2025 [63]
Genetic	ESR1 (rs2234693)	Estrogen receptor α	C allele is associated with greater flexibility	Better results on parallel bars and pommel horse	Kumagai et al., 2018 [67]
Genetic	CYP19A1 (rs936306)	Aromatase, conversion of androgens to estrogens	T allele contributes to tissue elasticity and flexibility	Greater mobility on apparatus requiring high range of motion	Hosono et al., 2015 [66]
Inflammatory biomarkers	IL-6, TNF-α	Pro-inflammatory cytokines	Released after physical stress, signal inflammation	Higher in non-specialized athletes after load	Mieszkowski et al., 2021 [70]
Bone marker	CTX (C-terminal telopeptide)	Marker of bone resorption	Increases after load in recreational athletes but not in elite gymnasts	Indicates a protective effect of long-term training on bone tissue	Mieszkowski et al., 2021 [70]
Muscle strength	WAnT test (Wingate)	Test of anaerobic power	Greater arm strength in gymnasts, no difference in leg strength	High specificity for upper-body strength in gymnastics	Mieszkowski et al., 2021 [70]
Vitamin D status	25(OH)D, 24,25(OH)_2_D_3_	Regulates bone and muscle metabolism	Lower values are associated with higher bone resorption (↑ CTX) and reduced bone protection	Low status may pose risk even in active athletes	Kumagai et al., 2022; [72] Hosono et al., 2015 [66]

Legend: ↑ indicates an increase compared to baseline values.

### 4.5. Biomechanical Specificities

Gymnastics is a sport that requires a high degree of biomechanical skill, involving precise coordination of body movements, strength, flexibility, and balance. The biomechanical specificities of gymnastic exercises play a crucial role in the occurrence of injuries. Certain movements, such as backflips or giant swings on the bars, place exceptional strain on specific joints and muscles, which can lead to strains, sprains, or even fractures [22].

One of the key factors contributing to injuries in gymnastics is improper technique during exercise performance, particularly when excessive demands are placed on gymnasts under competitive conditions [33]. Biomechanical misalignments, such as inefficient use of muscular strength or improper joint alignment, may also cause stress on particular body parts, especially the upper and lower extremities [32]. Specific injuries associated with the biomechanics of gymnastic exercises frequently include damage to the wrists, shoulders, knees, and lower back. The study by [22] reported that forearm injuries (36%) and ankle injuries (16.2%) are the most common among male gymnasts, while females are more prone to injuries of the hands and fingers, which may be explained by the different technical demands and biomechanical characteristics of their routines. In addition to these differences in injury patterns, gender-related variations in training load and physiological adaptation also appear to influence injury risk in artistic gymnastics. Female gymnasts typically experience higher relative training volumes at younger ages and perform a greater proportion of elements requiring flexibility and repetitive upper-limb support, which increases stress on the wrists and shoulders. In contrast, male gymnasts, who focus more on strength- and power-oriented apparatus such as rings and pommel horse, are exposed to higher joint loads in the upper body and greater cumulative stress on tendons and ligaments. These distinctions highlight the need for sex-specific monitoring of training intensity, recovery, and biomechanical technique to reduce injury incidence and optimize performance adaptations.

**Figure 2:**
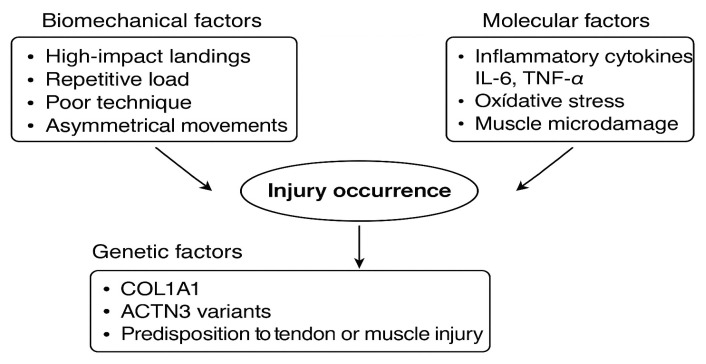
Multifactorial etiology of gymnastics-related injuries.

Biomechanical specificities also suggest that gymnastics training, which involves a high level of complexity, may cause increased stress on the musculoskeletal system. Excessive joint loading during the execution of elements that require high levels of strength, such as jumps or rotations, can result in micro-injuries that may develop into more serious conditions over time. The multifactorial nature of injuries in artistic gymnastics can be conceptualized through an integrated framework combining biomechanical, molecular, and genetic determinants (Figure 2).

Given these limitations, the need for further research aimed at improving both understanding and practice in the field of artistic gymnastics becomes evident. Despite existing data, there remains considerable scope for enhancing methodological approaches and deepening the understanding of cause-and-effect relationships between biomechanical loads and types of injuries. Future studies should include longitudinal designs, greater representation of both sexes and various age categories, as well as the application of modern methods of load monitoring, biomechanical analyses, and molecular profiling of athletes. Such a multidisciplinary approach may contribute to the development of personalized strategies for injury prevention and rehabilitation in gymnastics.

## 5. Conclusions

The conclusion of this study highlights the high incidence and specificity of injuries among gymnasts, particularly during periods of intensive growth, underscoring the need for early prevention and an individualized approach to the training process. Although this review addressed key aspects of biomechanical load, postural immaturity, and the importance of preventive measures, the limitations of the existing literature are reflected in the lack of longitudinal data and the insufficiently clear correlation between types of load and specific injuries across different age categories.

Furthermore, most available studies do not clearly distinguish between acute and chronic injuries, which further complicates the formulation of differentiated preventive and rehabilitative strategies. Future research is therefore recommended to include long-term monitoring of young gymnasts, detailed biomechanical analyses of movement, evaluation of the effects of different preventive programs, as well as consideration of individual factors such as body composition, flexibility, and previous injuries.

It is particularly important to emphasize the potential of genetic variants as key factors in predisposition for athletic success and injury risk. Polymorphisms in genes such as ACTN3, ESR1, and CYP19A1 are associated with specific components of physical fitness, explosive strength and flexibility, which are crucial for success in gymnastics. The integration of genetic profiling with molecular biomarkers of inflammation (IL-6, TNF-α, miRNA) may contribute to the development of personalized and more effective training and preventive protocols, incorporating a sex-specific approach.

The integration of a multidisciplinary approach, through the collaboration of coaches, physiotherapists, sports physicians, and researchers in molecular biology, can significantly contribute not only to the preservation of health but also to the optimization of athletic performance in young athletes.

Taking all of this into account, future research should aim toward the development of personalized and evidence-based strategies for injury prevention and treatment, integrating biomechanical, clinical, and molecular parameters with the goal of safeguarding health and ensuring athletic continuity.

## Data Availability

No new data were created or analyzed in this study. Data sharing is not applicable.

## Abbreviations

The following abbreviations are used in this manuscript:

ACL	Anterior Cruciate Ligament
ACTN3	Alpha-actinin-3
BMI	Body Mass Index
CYP19A1	Gene encoding aromatase enzyme
CTX	C-terminal telopeptide
ELISA	Enzyme-Linked Immunosorbent Assay
ESR1 gen	Estrogen Receptor 1
FIFA 11+	FIFA Injury Prevention Program
IL-6	Interleukin-6
miRNA	Mikro-Ribonukleinska kiselina
MLB	Major League Baseball
NBA	National Basketball Association
NFL	National Football League
qRT-PCR	Quantitative Real-Time Polymerase Chain Reaction
SNP	Single Nucleotide Polymorphism
TNF-α	Tumor Necrosis Factor alpha

## Appendix A

In Table A1, the scale used to assess the methodological quality of the studies included in this review is presented.

**Table A1:** Quality assessment scale of the included studies.

Authors	Selection (4)	Comparability of Groups (2)	Oucomes (3)	Total (9)
Westermann et al. (2015)	4	2	3	9
McLaren et al. (2015)	3	2	3	8
Saluan et al. (2015)	4	2	3	9
Guerra et al. (2016)	3	2	3	8
Rodríguez Camacho et al. (2016)	3	2	2	7
Goulart et al. (2016)	4	2	3	9
Boucher et al. (2017)	4	2	2	8
Trevithick et al. (2018)	4	2	3	9
Kox et al. (2018)	3	2	2	7
Ashwell et al. (2019)	3	2	2	7
Trevithick et al. (2019)	4	2	3	9
Paxinos et al. (2019)	4	2	3	9
Chandran et al. (2021)	4	2	3	9
Kruse et al. (2021)	4	2	2	8
Farì et al. (2021)	3	2	2	7
Tisano, Zynda et al. (2022)	4	2	2	8
Sastre Munar et al. (2022)	4	2	3	9
Pei et al. (2023)	4	2	2	8
Gram et al. (2025)	4	2	2	8

Legend: Selection: 1a. Representativeness of the exposed cohort, 2b. Selection of the non-exposed cohort, 3c. Ascertainment of exposure, 4d. Demonstration that the outcome of interest was not present at start of study; Comparability: 5e. Comparability of cohorts on the basis of design or analysis (adjusted for age), 6f. Comparability of cohorts on the basis of design or analysis (adjusted for any other factor); Outcome: 7g. Assessment of outcome, 8h. Sufficiency of follow-up duration for outcomes to occur, 9i. Adequacy of cohort follow-up; Quality Ratings: 0–3 (Poor), 4–6 (Fair), 7–9 (Good).
